# The role of physical activity in self-rated health in a region affected by mining dam collapse: Brumadinho Health Project

**DOI:** 10.3389/fpubh.2026.1760956

**Published:** 2026-04-21

**Authors:** Sergio Viana Peixoto, Mary Anne Nascimento-Souza, Juliana Vaz de Melo Mambrini, Josélia Oliveira Araújo Firmo, Maria Fernanda Lima-Costa

**Affiliations:** Rene Rachou Institute, Oswaldo Cruz Foundation, Belo Horizonte, Brazil

**Keywords:** epidemiology, human-made disasters, mining, physical activity, socioeconomic factors

## Abstract

**Introduction:**

Self-rated health is a robust measure of overall health status, encompassing an individual’s physical, mental, and social health. This multidimensional characteristic highlights the potential of this variable in studies conducted in regions affected by major disasters. This study assessed the prevalence of poor self-rated health and its association with sociodemographic factors, health behaviors, multimorbidity, and place of residence among adult residents of Brumadinho, Minas Gerais State, Brazil, following the collapse of a mining tailings dam.

**Materials and methods:**

It is a cross-sectional study based on baseline data from the Brumadinho Health Project, which was conducted in 2021 and included 2,771 individuals aged 18 years or older. The outcome variable was poor self-rated health, while the exploratory variables included sociodemographic characteristics, health behaviors, multimorbidity, and place of residence (area directly affected by the disaster; region with mining activity; unexposed). The association between exploratory variables and the outcome was evaluated using logistic regression.

**Results:**

The prevalence of poor self-rated health was 6.4% in the municipality’s population, being higher in the region directly affected by the tailings mud (12.7%). The likelihood of perceiving health as poor or very poor was higher among women, current smokers, and those with multimorbidity and lower among those who reported consuming alcoholic beverages one or more times per month. Engaging in physical activities at recommended levels reduced the likelihood of poor self-rated health, but only among the unexposed — residents in areas unaffected by mud and without mining activity (*p* interaction = 0.002).

**Discussion:**

These results demonstrate that the residential context modifies the effect of physical activity on self-rated health, such that residents in areas affected by tailings mud or with mining activity do not benefit from engaging in physical activity at recommended levels, at least regarding health perception. Greater efforts to improve environmental conditions may be necessary in regions impacted by major disasters or environmental degradation due to mining processes.

## 1 Introduction

Self-rated health reflects an individual’s perception of their overall health status and is a multidimensional measure that considers subjective, biological, psychosocial, and cultural aspects (1–3). It is widely used in epidemiological studies due to its ease of measurement and association with important health outcomes, including mortality (4, 5).

Overall, self-rated health is associated with sociodemographic conditions, the presence of diseases, and health behaviors. Poor self-rated health are more frequent among women, older individuals, those with lower education and income, and those with multimorbidity (6–9). Additionally, less healthy behaviors have been linked to poor self-rated health, such as smoking, unhealthy eating, and insufficient physical activity (8–10). Regarding alcohol consumption, there are reports of positive association (8, 11), negative association (9), or no association (12) with poor self-rated health.

In addition to individual factors, the physical and social environment is a determinant of self-rated health in various studies (13, 14). Residents in areas of greater economic deprivation (15), lower concentrations of green spaces (16), and higher pollution levels (17) tend to have poorer self-rated health. In this context, disasters that significantly alter the environment can worsen self-rated health, increasing the population’s vulnerability to adverse events. Generally, experiencing direct damage to one’s place of residence due to various disasters negatively affects health perception (18–21). However, there is evidence that physical activity is associated with better physical and mental health perception after traumatic events, suggesting it can be an effective measure for improving overall health in these scenarios (22).

In 2019, the tailings dam at the Córrego do Feijão mine, operated by the mining company Vale S.A., in Brumadinho, Minas Gerais State, Brazil, collapsed, causing significant environmental, economic, and social repercussions. It stands as one of the largest mining dam disasters in the world. The disaster resulted in 270 immediate deaths and released approximately 12 million cubic meters of tailings into the soil and the Paraopeba River basin, one of the most important water sources for the region’s public supply (23–25).

In this regard, studying self-rated health is particularly relevant, as this variable can aid in health planning and serve as a good indicator of the effectiveness of measures taken to mitigate the impact of major disasters (6, 26). Therefore, the objective of this study was to evaluate the prevalence of poor self-rated health and its association with sociodemographic factors, health behaviors, multimorbidity, and place of residence (area directly affected by the disaster; region with mining activity; unexposed) among adult residents of Brumadinho following the mining tailings dam collapse. Additionally, the study aimed to assess the potential modifying effect of the place of residence on the association between health behaviors and self-rated health.

## 2 Materials and methods

### 2.1 Data source

The Brumadinho Health Project is a prospective cohort study conducted in the municipality of Brumadinho, Minas Gerais. The primary objective of this research is to produce information about the population’s health conditions in the municipality following the disaster and in the subsequent years. More details can be found on the project’s website^1^ and another publication (27). The baseline information was collected between June and November 2021 and used in the analysis of the present study. The study received approval from the Research Ethics Committee of Fiocruz Minas (20814719.5.0000.5091) and was conducted in accordance with the principles outlined in the Declaration of Helsinki. All participants provided consent by signing the Informed Consent Form, and for underage participants, the Informed Consent Term was signed by caregivers, accompanied by the participant’s own informed assent.

### 2.2 Study area and population

**Figure 1:**
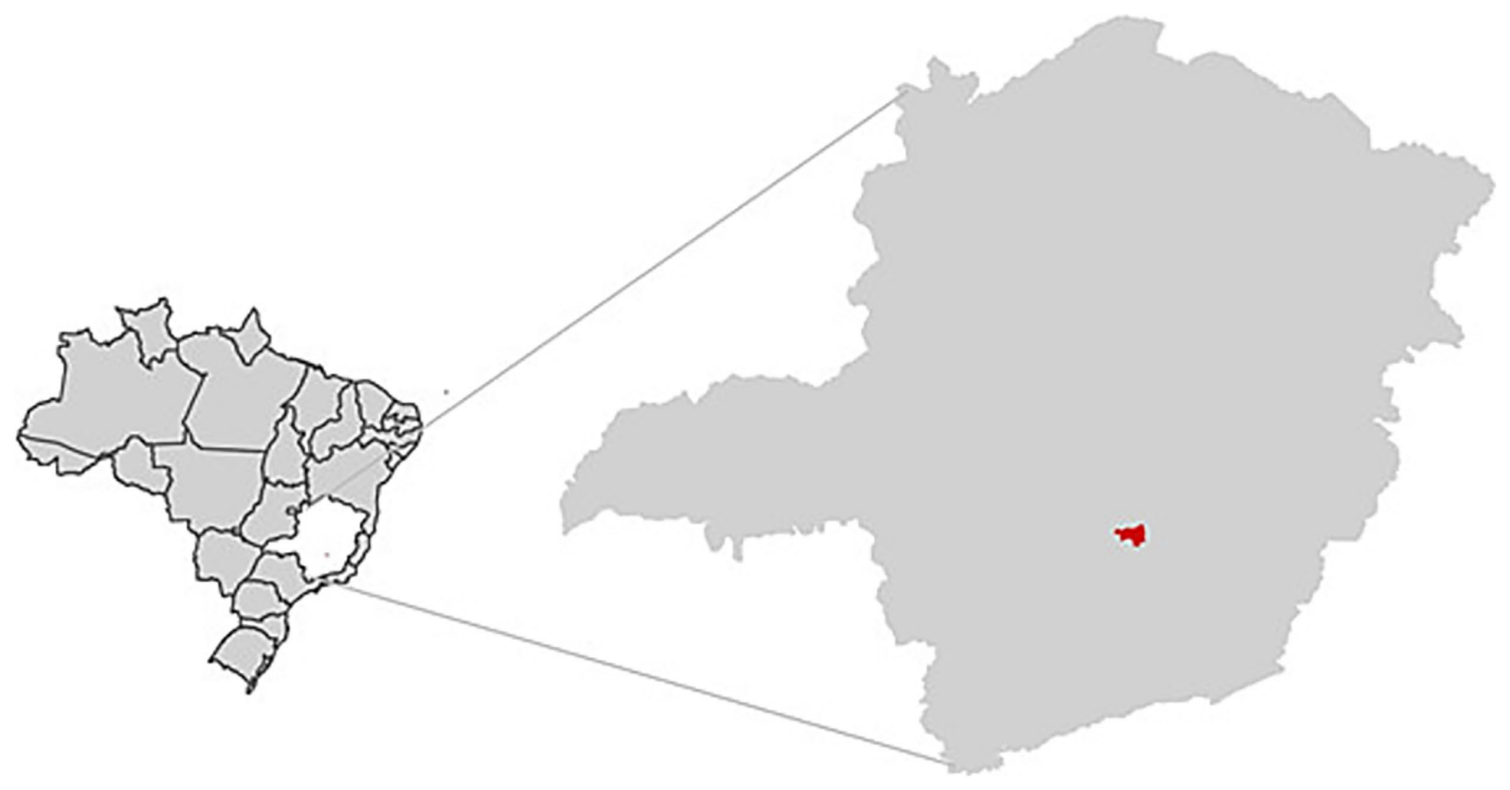
Location of Brumadinho (in red) in the Minas Gerais State and Brazil.

The municipality of Brumadinho is located in the Metropolitan Region of Belo Horizonte, the capital of the state of Minas Gerais, covering an area of 639.4 km^2^ (Figure 1). It has a population of 38,915 people and an infant mortality rate of 4.19 deaths per 1,000 live births, according to 2022 data (28).

The sampling plan was designed to represent the population residing in the municipality aged 12 years or older. This sample aimed to obtain information in three estimation domains: (1) the domain of those directly exposed to the collapse of the tailings dam at the Córrego do Feijão mine, which included the communities closest to the area affected by mud; (2) the domain of those residing in areas with mining activity; and (3) the domain of those not directly exposed to the tailings mud or mining activity (unexposed).

These sampling domains were composed of the census tracts of the municipality, a unit used by the Brazilian Institute of Geography and Statistics (IBGE) for data collection in surveys throughout the country. All households in regions considered exposed to mud or mining activity were included, as well as a random sample of households from the domain considered not directly exposed. A random sample of seven households was estimated through simple inverse sampling in all sectors that comprised the non-exposed area to ensure balance among the sampling domains.

For all domains, residents aged 12 years or older in the selected households were invited to participate in the research. A total of 1,446 households were visited, and 3,563 individuals aged 12 years or older were invited to participate, with an acceptance rate of 86.4%. Among the respondents, 2,802 were aged 18 years or older, of whom 2,771 had complete information for all variables considered in this study and were selected for the present analysis.

### 2.3 Variables and data collection

The information was collected through face-to-face household interviews with all selected household adult residents.

The dependent variable of this study was poor self-rated health, assessed by the question, “In general, how do you evaluate your health?” The response options were very good, fair, poor, and very poor. For the present analysis, the categories poor and very poor were combined and compared with those who responded very good, good, or fair.

The exploratory variables included sociodemographic characteristics, health behaviors, multimorbidity, and place of residence. In the first block, the sociodemographic characteristics considered were age group in years (18 to 39, 40 to 59, 60 or older), sex (male, female), and educational level (less than primary education, completed primary education, completed secondary education or higher).

Health behaviors included smoking, alcohol consumption, regular consumption of fruits and vegetables, and sufficient physical activity. Current smoking (no, yes) was assessed by the consumption of manufactured cigarettes at the time of the interview, whether daily or not, regardless of quantity. Alcohol consumption was evaluated based on the reported frequency of consumption, regardless of quantity (never, less than once a month, and once or more per month). Regular consumption of fruits and vegetables was assessed by reporting any amount of these foods at least five days a week (yes, no) (29). Physical activity was evaluated using the short version of the International Physical Activity Questionnaire (IPAQ), translated and validated for Brazil (30). This questionnaire considers physical activities performed the week before the interview for at least 10 min, including walking, moderate, and vigorous activities. Sufficient physical activity (no, yes) was defined as engaging in 150 min or more per week, with time spent on vigorous activities counted double (31).

Multimorbidity was assessed by self-reported medical diagnoses of two or more chronic conditions (32), evaluated through the question, “Has any doctor ever told you that you have…?”. The following diseases were considered: hypertension, diabetes, heart attack, angina, heart failure, stroke, asthma or asthmatic bronchitis, chronic bronchitis, emphysema or chronic obstructive pulmonary disease, arthritis or rheumatism, chronic kidney failure, cancer, chronic back problem, gastritis or ulcer, liver disease (except hepatitis), thyroid disease or problem, and depression.

Finally, the place of residence was characterized according to the sampling domains, with the population divided into (1) unexposed (random sample), (2) exposed to the tailings mud, and (3) exposed to mining activity.

### 2.4 Statistical analysis

For this study, the prevalence and corresponding 95% confidence interval for poor self-rated health were described. The exploratory variables analyzed were described according to the category of self-rated health, and the association with the outcome was evaluated using the Rao-Scott chi-square test. The magnitude of both crude and adjusted associations between self-rated health and the exploratory variables was estimated using logistic models (simple and multiple), with the calculation of odds ratios and 95% confidence intervals.

Interaction terms were included in the multiple model to evaluate the potential modifying effect of place of residence on the association between health behaviors and self-rated health. Significant interaction was observed only between place of residence and physical activity, and these results were illustrated by a predicted probability graph.

All analyses were conducted using Stata 18.0 (StataCorp LLC, College Station, TX), accounting for sample weights and the effect of the sampling design through the svy command, which is appropriate for complex samples.

## 3 Results

Among the 2,771 participants, 6.4% (95% CI: 5.2–7.7) perceived their health as poor or very poor. This prevalence was 12.9% (95% CI: 10.7–15.4) in the area directly affected by the tailings mud and 8.3% (95% CI: 6.2–11.1) among residents in areas with mining activity.

Table 1 shows the sample characteristics. Of the total, 23.4% were aged 60 years or older, 49.1% were men, and 35.4% had low educational attainment (less than primary education). Among health behaviors, 17.2% were current smokers at the time of the interview, 39.9% consumed alcoholic beverages once or more per month, 32.9% had regular consumption of fruits and vegetables, and 69.7% engaged in physical activity at sufficient levels. Two or more chronic conditions were reported by 33.4% of participants, and the majority of the sample (95.3%) resided in areas unaffected by the dam collapse or mining activity. Except for regular consumption of fruits and vegetables, all variables showed a significant association with health perception in this analysis (*p* < 0.05).

**Table 1:** Distribution of sociodemographic characteristics, health behaviors, multimorbidity, and place of residence of the total study population and according to poor self-rated health.

Variables		Poor self-rated health		
	Total	No	Yes	*p*-value*
Age group (years)				<0.001
18 to 39	37.2	38.1	23.3	
40 to 59	39.4	39.7	35.5	
60 or more	23.4	22.2	41.2	
Sex				
Male	49.1	50.3	31.1	0.001
Female	50.9	49.7	68.9	
Education				
Less than primary education	35.4	33.9	58.8	<0.001
Completed primary education	17.8	18.0	15.3	
Completed secondary education or higher	46.8	48.1	25.9	
Current smoking				0.031
No	82.8	83.4	74.6	
Yes	17.2	16.6	25.4	
Alcohol consumption				0.001
Never	48.9	47.9	63.9	
Less than once a month	11.2	11.0	14.7	
Once or more per month	39.9	41.1	21.4	
Regular consumption of fruits and vegetables^a^				0.059
No	67.1	66.6	75.9	
Yes	32.9	33.4	24.1	
Sufficient physical activity^b^				<0.001
No	30.3	28.3	60.2	
Yes	69.7	71.7	39.8	
Multimorbidity^c^				<0.001
No	66.6	69.1	29.6	
Yes	33.4	30.9	70.4	
Place of residence				
Unexposed	95.3	95.6	91.7	<0.001
Exposed to tailings mud	3.0	2.8	6.1	
Exposed to mining activity	1.7	1.6	2.2	

Brumadinho Health Project, Minas Gerais, Brazil, 2021. *Rao-Scott chi-square test. Values are expressed as percentages.

a Consumption on at least five days of the week.

b At least 150 min/week, including walking, moderate, or vigorous activities.

c Two or more diseases among 16 chronic diseases evaluated.

The magnitude of the crude associations is described in Table 2. The likelihood of poor self-rated health was higher among older individuals, women, current smokers, those with two or more chronic conditions, and residents in the region affected by the dam collapse. Lower likelihoods were observed among those with higher education levels, those who consumed alcohol once or more per month, and those who engaged in physical activity at sufficient levels.

**Table 2:** Crude analysis of the association between sociodemographic characteristics, health behaviors, multimorbidity, place of residence, and poor self-rated health.

Variables	Crude OR* (95% CI)
Age group (years)	
18 to 39	1.00
40 to 59	1.46 (0.83–2.57)
60 or more	3.05 (1.72–5.40)
Sex	
Male	1.00
Female	2.24 (1.37–3.67)
Education	
Less than primary education	1.00
Completed primary education	0.49 (0.27–0.90)
Completed secondary education or higher	0.31 (0.18–0.53)
Current smoking	
No	1.00
Yes	1.71 (1.04–2.81)
Alcohol consumption	
Never	1.00
Less than once a month	1.00 (0.52–1.95)
Once or more per month	0.39 (0.23–0.67)
Regular consumption of fruits and vegetables^a^	
No	1.00
Yes	0.63 (0.39–1.02)
Sufficient physical activity^b^	
No	1.00
Yes	0.26 (0.17–0.41)
Multimorbidity^c^	
No	1.00
Yes	1.67 (1.49–1.87)
Place of residence	
Unexposed	1.00
Exposed to tailings mud	2.28 (1.67–3.11)
Exposed to mining activity	1.40 (0.95–2.07)

Brumadinho Health Project, Minas Gerais, Brazil, 2021. *Crude odds ratio (95% confidence interval).

a Consumption on at least five days of the week.

b At least 150 min/week, including walking, moderate, or vigorous activities.

c Two or more diseases among 16 chronic diseases evaluated.

In the multiple analysis, interactions between health behaviors and region of residence were tested. Only the interaction between region and physical activity was significant (*p* = 0.002). Table 3 describes the adjusted associations. The chance of poor self-rated health was higher among women (OR: 2.06; 95% CI: 1.18–3.60), current smokers (OR: 1.98; 95% CI: 1.13–3.46), and those with two or more chronic conditions (OR: 4.11; 95% CI: 2.41–7.03). It was lower among those who reported consuming alcoholic beverages once or more per month (OR: 0.55; 95% CI: 0.31–0.96).

**Table 3:** Adjusted analysis of the association between sociodemographic characteristics, health behaviors, multimorbidity, place of residence, and poor self-rated health.

Variables	Adjusted OR (95% CI)*
Age group (years)	
18 to 39	1.00
40 to 59	0.80 (0.40–1.60)
60 or more	1.29 (0.63–2.66)
Sex	
Male	1.00
Female	2.06 (1.18–3.60)
Education	
Less than primary education	1.0
Completed primary education	0.83 (0.43–1.62)
Completed secondary education or higher	0.52 (0.26–1.04)
Current smoking	
No	1.00
Yes	1.98 (1.13–3.46)
Alcohol consumption	
Never	1.00
Less than once a month	1.62 (0.79–3.32)
Once or more per month	0.55 (0.31–0.96)
Regular consumption of fruits and vegetables^a^	
No	1.00
Yes	0.58 (0.33–1.01)
Multimorbidity^b^	
No	1.00
Yes	4.11 (2.41–7.03)

Brumadinho Health Project, Minas Gerais, Brazil, 2021. *Odds ratio (95% confidence interval) adjusted for all variables listed in the table, as well as place of residence, physical activity, and the interaction term between these two variables.

a Consumption on at least five days of the week.

b Two or more diseases among 16 chronic diseases evaluated.

Engaging in physical activity at sufficient levels reduced the chance of perceiving health as poor or very poor, but only among residents in the unexposed regions (OR: 0.30; 95% CI: 0.18–0.50). This effect was not significant among residents in the area directly affected by mud (OR: 1.07; 95% CI: 0.67–1.71) or among those residing in areas with mining activity (OR: 0.77; 95% CI: 0.37–1.58) (data not shown in Table 3).

**Figure 2:**
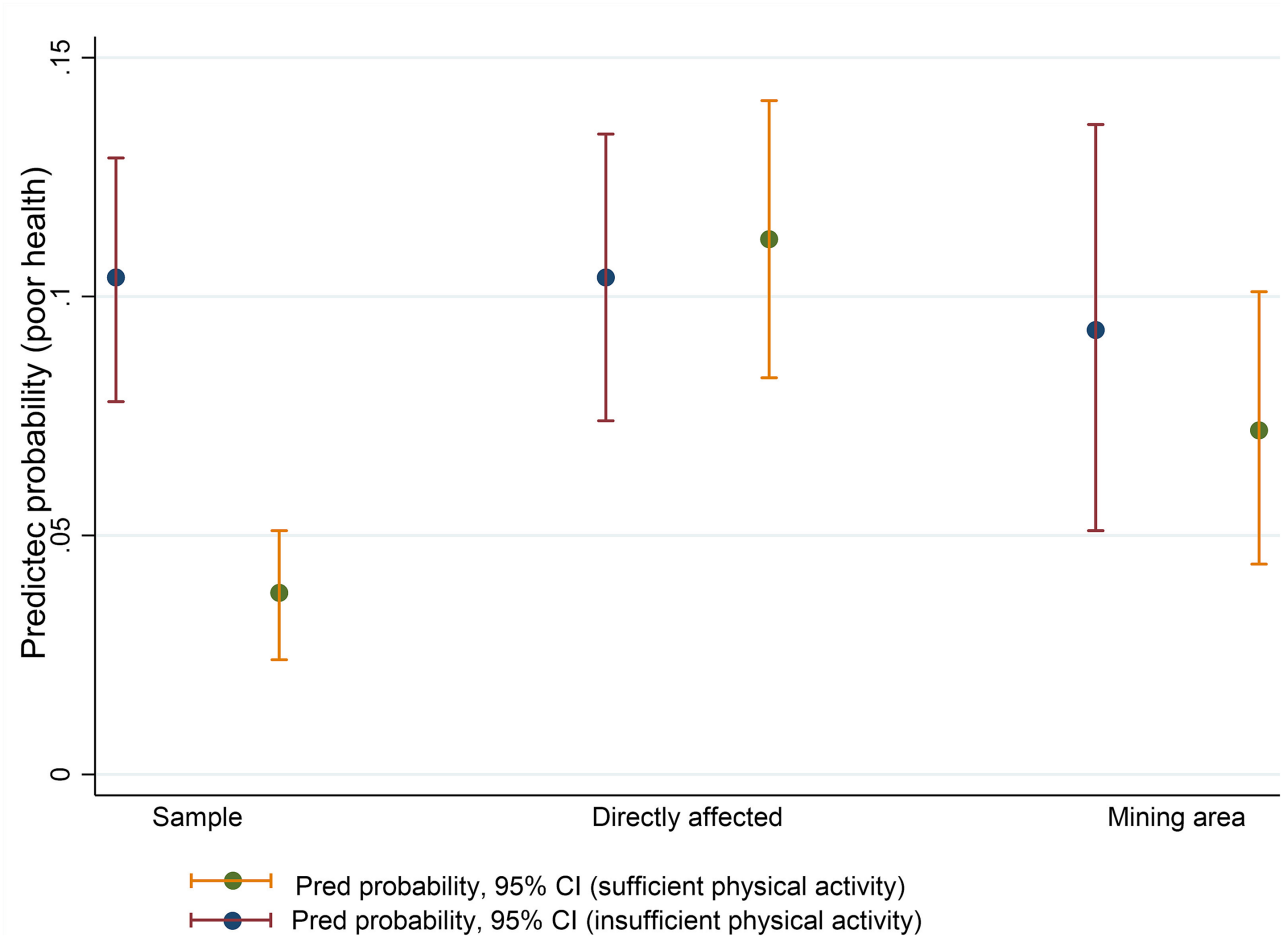
Interaction between the region of residence and sufficient physical activity.

Figure 2 illustrates the interaction between the place of residence and sufficient physical activity. For residents in the area unaffected by the dam collapse and not exposed to mining activity, those who engaged in sufficient physical activity had a lower predicted probability of reporting poor self-rated health than those who were insufficiently active. This effect was not observed in the other regions.

## 4 Discussion

The results of this study showed that the prevalence of poor self-rated health was twice as high in the area directly affected by mud (12.7%) compared to residents in unexposed areas (6.4%). After adjusting for all variables, the chance of perceiving health as poor or very poor was higher among women, current smokers, and those with multimorbidity and lower among those who reported consuming alcoholic beverages once or more per month. Additionally, engaging in physical activity at recommended levels reduced the chance of poor self-rated health, but only among residents in areas unaffected by mud and without mining activity.

The proportion of adults rating their health as poor varies significantly across populations. In national samples, this proportion was 3.6% in Canada (33) and had a median of 4.0% in the United States, with wide variation between states – 2.5 to 8.4% (34). Higher values were observed in Spain (9.4%) (35), South Africa (17.0%) (36), and Iran (17.2%) (37). In Brazil, data from the National Health Survey—“Pesquisa Nacional de Saúde” (2019) showed that 5.8% of the adult population rated their health as poor or very poor (38), a value similar to that observed in Brumadinho (6.4%). However, the directly affected area recorded a higher prevalence (12.7%). This variation reflects the diversity of factors influencing self-rated health, including sociodemographic, psychosocial, and cultural aspects, as well as physical and mental health (3).

The higher prevalence of poor self-rated health among residents in the areas directly affected by the tailings dam mud may be related to environmental degradation and poorer socioeconomic conditions. A study conducted in three localities in the Aral Sea region, Central Asia, an area with significant environmental degradation due to irrigation projects for agriculture and pesticide use, reported a prevalence of poor health self-perception of 12.0%. This proportion was higher in the locality closest to the Aral Sea (14.0%) and lower in the more distant region (9.0%) (39). The significant environmental degradation observed in the directly affected areas of Brumadinho (23) may explain the poor self-rated health in these areas, considering the importance of the environment for this evaluation (14, 16, 40). Additionally, some disaster consequences, such as reduced social cohesion, stressful experiences, and damage to homes, have been related to poor self-rated health (19, 21, 41), reinforcing the vulnerability of residents in these regions.

In Brumadinho, higher chances of poor self-rated health were observed among women, current smokers, and those reporting two or more chronic conditions. This profile is consistent with that observed in previous studies conducted in regions that have experienced (20, 41, 42) or have not experienced (6, 8, 9) a disaster. However, the association between health perception and alcohol consumption is inconsistent across studies (8, 9, 12). In Brumadinho, consuming alcoholic beverages once or more per month reduced the chance of reporting poor self-rated health, which may be related to reverse causality, considering that individuals with poorer health conditions are less likely to consume alcoholic beverages (43).

Physical activity has been consistently associated with better self-rated health in different contexts. This effect has been observed among Canadian adults (10), Spaniards (44), Estonians (43), and residents of Eastern countries (45, 46). Similarly, in Brazil, an analysis of telephone survey (Vigitel) data showed that adults who walked more than 150 min during leisure time had a higher chance of perceiving their health as good (47). This result was also observed among Japanese adults three years after an earthquake, demonstrating that physical activity was related to better health perception (48). In Brumadinho, two years after the dam collapse, sufficient physical activity levels reduced the chance of poor self-rated health, but only among residents in areas unaffected by mud or without mining activity.

This interaction between physical activity and place of residence may reflect the importance of the environment in determining health perception in the context of Brumadinho. As previously mentioned, the social and built environment significantly influences health perception (16, 17, 40). Although physical activity can promote better physical and mental health after traumatic events (22), in Brumadinho, this behavior did not reduce the chance of reporting poor self-rated health among residents in areas degraded by the disaster or by mining activity.

As the perception of the environment is also important for the practice of physical activity (49), these findings highlight the need to direct efforts toward areas affected by the disaster and mining activity. Creating a favorable environment for physical activity can enhance health perception among these populations.

This study has some limitations that should be considered. Data collection for this research occurred two years after the tailings dam collapse, which does not capture the immediate profile of the population following the disaster. Additionally, the cross-sectional nature of the study does not allow for establishing temporal relationships between the outcome and explanatory variables, raising the possibility of reverse causality. Finally, the use of self-reported questions may have overestimated or underestimated the frequencies of the variables of interest, although it is a widely used strategy in epidemiological studies. On the other hand, the analyzed data comes from the baseline of a prospective population-based study conducted with a representative sample of the population residing in Brumadinho after a major disaster. Information was collected by trained interviewers using standardized questionnaires, with rigorous supervision of this process.

Self-rated health is a multidimensional measure widely used in epidemiological studies conducted in various contexts, including the evaluation of living and health conditions of populations affected by major disasters. In Brumadinho, after the collapse of the mining tailings dam, higher chances of reporting poor self-rated health were observed among women, current smokers, and those reporting two or more chronic conditions. In comparison, lower chances were observed among those who reported consuming alcoholic beverages once or more per month. However, engaging in sufficient physical activity level reduced the likelihood of poor self-rated health only in the region unaffected by the disaster and not impacted by mining activity. This finding demonstrates that residing in areas affected by mud or mining activity modifies the effect of physical activity, which does not provide the expected benefit for self-rated health in these regions. This result highlights the importance of the residential context and the need for greater efforts to improve health perception and, consequently, related outcomes in the most impacted regions.

## Data Availability

The raw data supporting the conclusions of this article will be made available by the authors, without undue reservation.

## Footnotes

1
http://www.minas.fiocruz.br/saudebrumadinho/
